# Modifiable Lifestyle Factors in Cardiovascular Disease Prevention: Current Evidence and Future Directions

**DOI:** 10.3390/nu18183104

**Published:** 2026-09-21

**Authors:** Patrick Benjamin, Luc Djoussé

**Affiliations:** 1 VA Boston Healthcare System, 1620 Tremont Street, 3rd Floor, Boston, MA 02120, USA; ldjousse@bwh.harvard.edu; 2 Mass General Brigham, Boston, MA 02115, USA; 3 Massachusetts Veterans Epidemiology Research and Information Center (MAVERIC), Boston, MA 02130, USA; 4 Geriatric Research, Education, and Clinical Center (GRECC), VA Boston Healthcare System, Boston, MA 02130, USA; 5 Harvard T H Chan School of Public Health, Boston, MA 02115, USA

**Keywords:** cardiovascular disease, prevention, lifestyle, smoking, physical activity, diet, sleep

## Abstract

Cardiovascular disease (CVD) remains the leading cause of morbidity and mortality worldwide despite substantial advances in pharmacologic and interventional therapies. Modifiable lifestyle behaviors are major determinants of cardiovascular risk and essential targets for primary and secondary prevention. This narrative review summarizes contemporary evidence on smoking cessation, physical activity and exercise, nutrition, alcohol consumption, sleep health, sedentary behavior, and integrated lifestyle strategies. Evidence from randomized trials supports cardiovascular risk-factor and event reduction with selected dietary and exercise interventions (i.e., Mediterranean and Dietary Approaches to Stop Hypertension (DASH)), whereas many estimates for sleep, sedentary behavior, alcohol and composite lifestyle scores derive from observational studies and should be interpreted as associations rather than causal effects. Conversely, unhealthy dietary habits, tobacco use, excessive alcohol consumption, poor sleep quality, and prolonged sedentary behavior contribute to endothelial dysfunction, inflammation, metabolic dysregulation, and accelerated atherosclerosis and subsequent increased risk of CVD. Emerging evidence further underscores the importance of considering lifestyle behaviors collectively rather than individually, as their effects may be synergistic. Comprehensive lifestyle modification remains one of the most effective and cost-effective approaches to reducing cardiovascular risk and should be integrated into routine clinical practice and public health initiatives.

## 1. Introduction

Cardiovascular disease (CVD) remains the leading cause of death worldwide, accounting for approximately 19.4 million deaths and affecting an estimated 612 million individuals globally in 2021, with CVD deaths increasing by 18% since 2010 [1,2]. Despite substantial advances in preventive pharmacotherapy, revascularization strategies, and heart failure management, CVD continues to impose a considerable clinical and economic burden [2,3,4,5]. In the United States, approximately 48.9% of adults aged ≥20 years—representing an estimated 130.6 million individuals—have some form of CVD, with average annual direct and indirect healthcare costs estimated at $414.7 billion [2].

Although pharmacologic therapies have reduced cardiovascular mortality, a substantial proportion of cardiovascular events remain preventable. Lifestyle behaviors—including smoking, physical inactivity, unhealthy dietary habits, heavy alcohol consumption, poor sleep, and prolonged sedentary behavior—contribute significantly to the development of coronary heart disease, stroke, heart failure, and cardiovascular mortality [4,5,6]. Importantly, these behaviors often coexist and cumulatively affect cardiovascular health. Given the rapidly expanding evidence base and increasing emphasis on lifestyle medicine, an updated synthesis of the major modifiable lifestyle determinants of cardiovascular health is warranted.

**Figure 1:**
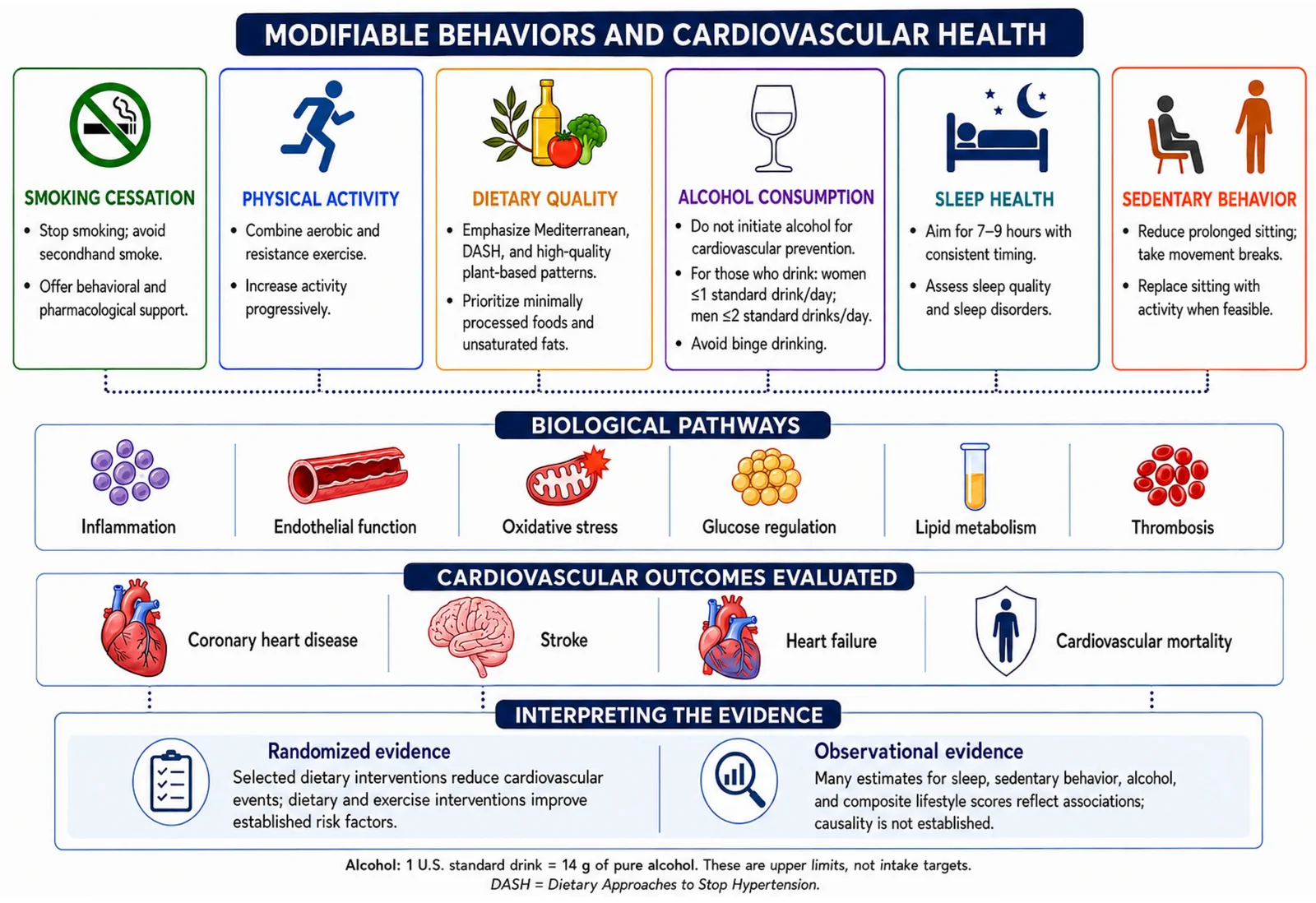
Modifiable lifestyle factors may influence cardiovascular health through multiple biological pathways. The strength and type of supporting evidence vary across lifestyle domains and outcomes.

This review summarizes current evidence on major modifiable health behaviors relevant to cardiovascular disease prevention, distinguishes observational associations from intervention effects, and discusses future opportunities to improve cardiovascular health at individual and population levels. Major lifestyle factors and shared biological mechanisms are summarized in Figure 1.

## 2. Evidence Identification and Selection

This opinion article used a non-systematic narrative literature search to provide a broad, balanced synthesis rather than a formal systematic review. We searched PubMed/MEDLINE, Google Scholar, and major professional-society guideline and scientific-statement repositories for English-language evidence available through 31 August 2026. Searches combined terms related to cardiovascular disease with diet or dietary patterns, smoking or tobacco, physical activity, exercise, resistance training, cardiorespiratory fitness, sedentary behavior, alcohol, sleep, body composition, and Life’s Essential 8. Priority was given to randomized controlled trials and their meta-analyses for intervention effects, followed by systematic reviews/meta-analyses of prospective cohorts, large prospective cohort studies, and contemporary guidelines or scientific statements. Observational studies were included when randomized evidence for long-term clinical outcomes was unavailable. Reference lists of key reviews and guidelines were also examined for relevant studies. Study selection was narrative and not performed according to PRISMA; therefore, this review should not be interpreted as an exhaustive systematic review. During revision, quantitative estimates were checked against the cited original source for study population, exposure or intervention, comparator, outcome, effect measure, and confidence interval, and causal language was reserved for evidence capable of supporting causal inference.

## 3. Dietary Patterns and Cardiovascular Disease

Contemporary dietary guidelines increasingly emphasize overall dietary patterns because these patterns better reflect habitual food intake and capture synergistic interactions among foods. Dietary patterns provide a more comprehensive assessment of cardiovascular risk than individual foods and/or nutrients because foods are consumed together and produce complementary biological effects. Contemporary evidence supports dietary patterns rich in minimally processed foods, whole grains, healthy fats, legumes, fish, and nuts, while limiting refined carbohydrates, added sugars, sodium, and processed meats [7,8,9,10]. These diets improve blood pressure, lipid profiles, insulin sensitivity, endothelial function, and systemic inflammation, thereby reducing cardiovascular risk [7,9].

## 4. Mediterranean Diet

The Mediterranean diet is among the most extensively studied dietary patterns for cardiovascular prevention. Characterized by high intake of fruits, vegetables, legumes, whole grains, olive oil, nuts, and fish, it has consistently been linked to lower risks of cardiovascular events and mortality [11,12]. In the PREDIMED trial, a Mediterranean diet supplemented with extra-virgin olive oil (HR 0.69; 95% CI 0.53–0.91) or mixed nuts (HR 0.72; 95% CI 0.54–0.95) reduced the risk of major adverse cardiovascular events by about 30% compared with a low-fat diet for extra-virgin oil and [10]. A meta-analysis of prospective cohort studies found that each two-point increase in Mediterranean-diet adherence score was associated with a 9% lower risk of cardiovascular mortality (RR 0.91; 95% CI 0.87–0.95) [12].

## 5. DASH Dietary Pattern

The Dietary Approaches to Stop Hypertension (DASH) diet emphasizes fruits, vegetables, whole grains, legumes, nuts, and low-fat dairy while limiting sodium, saturated fat, and added sugars. In the original DASH trial, systolic blood pressure decreased by 5.5 mmHg and diastolic blood pressure by 3.0 mmHg compared with the control diet, with even greater reductions among individuals with hypertension [13,14]. Subsequent prospective cohort studies have associated greater adherence to the DASH diet with lower risks of coronary heart disease and stroke in women and a 22% lower rate of heart failure in men [15,16].

## 6. Plant-Based Dietary Patterns

High-quality plant-based dietary patterns have attracted growing attention for their cardiovascular benefits [17,18,19]. Importantly, observed cardiovascular outcomes depend on diet quality rather than on simply reducing animal products. Among 148,506 participants in the Million Veteran Program who were free of diabetes, CVD, and cancer at baseline, each 10-point increment in the overall Plant-Based Diet Index was associated with a 17% lower risk of total CVD (HR 0.83; 95% CI 0.78–0.88), and each 10-point increment in the healthful Plant-Based Diet Index was associated with a 16% lower risk (HR 0.84; 95% CI 0.80–0.88). Conversely, the unhealthful Plant-Based Diet Index was associated with higher total CVD risk (HR 1.07 per 10-point increment; 95% CI 1.03–1.12) [20]. Because these estimates derive from prospective observational data, they demonstrate association rather than a randomized intervention effect [18].

## 7. Dietary Quality and Food Components

Beyond overall dietary patterns, several food components independently contribute to cardiovascular health. Meta-analyses show that each 90-g/day increase in whole-grain intake is associated with about a 19% lower risk of coronary heart disease and a 22% lower risk of cardiovascular disease [21]. Replacing saturated fats with polyunsaturated fats lowers LDL cholesterol and reduces cardiovascular events, whereas diets high in sodium, sugar-sweetened beverages, and ultra-processed foods are consistently linked to higher risks of hypertension, obesity, diabetes, and cardiovascular disease [22,23,24]. A recent umbrella review reported that individuals with the highest consumption of ultra-processed foods had about a 17–50% greater risk of cardiovascular disease and cardiovascular mortality, depending on the outcome examined [25].

Overall, the accumulated evidence supports prioritizing dietary quality over isolated nutrients or foods. Randomized evidence supports favorable effects of Mediterranean and DASH dietary patterns on cardiovascular events or major risk factors, while many long-term estimates for other dietary patterns remain observational [7,8,9,10,11,12,13,14,15,16,17,18,19,21,22,26].

## 8. Smoking and Cardiovascular Disease

Smoking remains one of the most important preventable causes of cardiovascular disease worldwide [27,28,29,30,31]. Cigarette smoke promotes endothelial dysfunction, oxidative stress, inflammation, thrombosis, and accelerated atherosclerosis [32]. Meta-analytic evidence demonstrates a strong dose-response relationship between smoking and cardiovascular risk. Aune et al., in a systematic review, found that current smokers have more than a threefold higher risk of sudden cardiac death than never-smokers (RR 3.08, 95% CI 2.46–3.84) [30]. Smoking also substantially increases the risk of coronary artery disease, stroke, peripheral arterial disease, and heart failure [27,28,29,30,31]. A meta-analysis of 14 studies found that smokers had a significantly higher risk of stroke than non-smokers (OR 1.61, 95% CI 1.34–1.93) [33]. Similarly, a dose–response meta-analysis of prospective studies found that heart failure risk decreased with increasing time since smoking cessation, with an estimated 28% lower risk at 30 years after cessation than with current smoking (RR 0.72; 95% CI 0.57–0.90) [34]. Because much of the long-term outcome evidence is observational, the exact magnitude and time course vary by population; nevertheless, smoking cessation remains a cornerstone of cardiovascular prevention.

Passive smoking also contributes significantly to the cardiovascular burden. Exposure to secondhand smoke has been associated with a 27% higher risk of ischemic heart disease compared with no exposure (RR 1.27, 95% CI 1.10–1.48) [35]. These findings reinforce smoking prevention and cessation as cornerstone strategies for preventing cardiovascular disease.

## 9. Physical Activity and Cardiovascular Disease

Physical activity is any bodily movement that increases energy expenditure, whereas exercise is planned and structured physical activity. Cardiorespiratory fitness is a physiologic attribute influenced by activity, genetics, age, and health status. Sedentary behavior is considered separately because a person can meet exercise targets yet still accumulate prolonged sedentary time. Current guidelines recommend at least 150 min of moderate-intensity aerobic activity, 75 min of vigorous activity, or an equivalent combination each week, together with muscle-strengthening activity on at least 2 days per week [4,5,36,37]. These targets should not be interpreted as a threshold below which activity has no value: dose-response analyses show that the largest relative gains often occur when inactive individuals undertake even modest amounts of activity [37,38,39,40,41].

Regular aerobic exercise improves endothelial function, blood pressure, insulin sensitivity, lipid profiles, autonomic balance, and cardiorespiratory fitness [42,43]. Resistance training complements aerobic activity by preserving or increasing skeletal muscle mass and strength and can improve blood pressure, glycemia, lipid profiles, and body composition [37]. Cardiorespiratory fitness should therefore be distinguished from physical activity itself, although improving fitness is an important outcome of appropriately prescribed exercise.

Prospective cohort evidence demonstrates a graded inverse association between non-occupational physical activity and cardiovascular events and mortality [37,38,39,40,41]. A recent dose-response meta-analysis of 196 articles representing 94 cohorts and more than 30 million participants found meaningful risk reductions at activity volumes below guideline targets, with additional benefit as activity increased [36]. These observational estimates complement randomized evidence showing favorable effects of exercise on established cardiovascular risk factors. Resistance training is also an important component: the American Heart Association notes epidemiologic associations with lower CVD risk and randomized evidence of improvement in several cardiovascular risk factors [37].

The practical message is therefore to reduce inactivity, progressively increase sustainable movement, and combine aerobic and resistance exercise when feasible. Observational studies have examined whether the time of day of activity affects cardiovascular risk, but current evidence is insufficient to recommend morning rather than later exercise for cardiovascular prevention; total activity, consistency, safety, and adherence remain the priorities [44].

## 10. Alcohol Consumption and Cardiovascular Disease

The relationship between alcohol consumption and cardiovascular disease remains complex and controversial. Long-term cardiovascular outcomes have not been tested in a sufficiently powered randomized trial. Some prospective observational studies and meta-analyses have reported lower rates of selected coronary or heart-failure outcomes among people reporting low-to-moderate intake compared with abstainers [45,46,47]. However, other large prospective and genetic analyses challenge a cardioprotective interpretation and show increasing vascular risk with greater alcohol exposure [48,49]. These apparently discordant findings highlight the limitations of using observational drinking categories to infer a causal preventive effect.

Observational estimates are vulnerable to residual socioeconomic and behavioral confounding, exposure misclassification, and ‘sick-quitter’ bias, in which former drinkers with illness are included among abstainers. Average intake also incompletely captures drinking patterns, binge exposure, beverage type, and changes over time. Consequently, associations between low reported intake and lower cardiovascular event rates should not be interpreted as evidence that alcohol itself prevents cardiovascular disease.

Alcohol has heterogeneous cardiovascular and non-cardiovascular effects. Higher intake is associated with hypertension, atrial fibrillation, cardiomyopathy, hemorrhagic stroke, and heart failure, and alcohol increases the risk of several cancers even at relatively low exposure.

## 11. Sleep Health and Cardiovascular Disease

Sleep health is multidimensional and includes duration, quality, regularity, timing, efficiency, daytime alertness, and sleep disorders; sleep duration alone does not fully characterize cardiovascular sleep health [50]. In a meta-analysis of prospective cohorts, short sleep was associated with higher risk of coronary heart disease (RR 1.48; 95% CI 1.22–1.80) and stroke (RR 1.15; 95% CI 1.00–1.31) but was not significantly associated with total CVD (RR 1.03; 95% CI 0.93–1.15). Long sleep was associated with CHD (RR 1.38; 95% CI 1.15–1.66), stroke (RR 1.65; 95% CI 1.45–1.87), and total CVD (RR 1.41; 95% CI 1.19–1.68) [51]. These are observational associations and do not establish that sleep duration itself causes cardiovascular events.

Short or disrupted sleep may influence sympathetic activation, inflammation, endothelial function, blood pressure, and glucose metabolism [52]. Obstructive sleep apnea is independently relevant to hypertension, atrial fibrillation, coronary disease, and heart failure [53]. Conversely, prolonged sleep can be a marker of underlying illness, frailty, depression, low physical activity, sleep fragmentation, or other confounding conditions [54]. Clinical assessment should therefore consider sleep quality, regularity, timing, and sleep disorders rather than treating duration in isolation.

## 12. Sedentary Behavior and Cardiovascular Disease

Sedentary behavior is distinct from physical inactivity and refers to low-energy waking behavior in a sitting, reclining, or lying posture. Meta-analytic observational evidence links greater sedentary time to adverse cardiovascular outcomes, even after adjusting for physical activity, although residual confounding remains possible [55,56]. In the Biswas et al. meta-analysis, high sedentary time was associated with higher cardiovascular mortality (HR 1.18; 95% CI 1.11–1.26) [55]. Intervention trials that replace sitting with standing or light activity primarily show changes in intermediate cardiometabolic outcomes rather than proven reductions in cardiovascular events. Thus, reducing prolonged sitting and incorporating regular movement breaks is reasonable.

## 13. Life’s Essential 8 and Cardiovascular Health

Life’s Essential 8 (LE8) is a cardiovascular health framework rather than a list of eight equivalent lifestyle behaviors. It comprises four health behaviors—diet, physical activity, nicotine exposure, and sleep—and four health factors—body mass index/body weight, blood pressure, blood glucose, and blood lipids [50]. The clinical factors are important intermediate measures of cardiovascular health, but they should not be used interchangeably with lifestyle behaviors. The overall score is useful because it integrates behavioral and clinical domains into a common cardiovascular health construct.

Recent observational evidence demonstrates graded associations between higher LE8 scores and lower cardiovascular risk. A 2025 systematic review and meta-analysis including 34 observational studies and approximately 1.79 million participants found that higher versus lower LE8 scores were associated with lower risks of CVD (HR 0.47; 95% CI 0.39–0.56), all-cause mortality (HR 0.54; 95% CI 0.43–0.69), and cardiovascular mortality (HR 0.37; 95% CI 0.26–0.52), although substantial between-study heterogeneity was present [57]. These findings support LE8 as a risk and health construct but do not establish that changing the composite score by intervention produces an equivalent causal reduction in events. Body weight also warrants nuance. BMI is pragmatic for population assessment but does not distinguish visceral adiposity from lean mass. Particularly in older adults, prevention should consider waist/central adiposity, preservation of skeletal muscle, strength, and physical function in addition to weight alone [58]. Resistance exercise can improve or preserve muscle mass and strength while favorably affecting body composition and cardiometabolic risk factors [37]. Thus, weight management should emphasize excess adiposity reduction without unnecessary loss of lean tissue rather than pursuing weight loss as an isolated target.

## 14. Combined Effects of Lifestyle Factors

Lifestyle behaviors rarely occur in isolation, and prospective cohorts show graded associations between more favorable behaviors and lower cardiovascular risk. In the Physicians’ Health Study, men with four or more favorable lifestyle factors had a lower lifetime risk of heart failure than those with none (10.1% vs. 21.2%), but this was an observational association [59]. In a Finnish cohort, increasing numbers of favorable lifestyle factors were likewise associated with progressively lower heart-failure risk [60]. Composite lifestyle scores should therefore be interpreted as markers of clustered behaviors rather than as randomized multicomponent interventions.

Similar observational studies demonstrate that favorable lifestyle patterns are associated with lower coronary risk, even among people with high genetic susceptibility [61]. Collectively, these studies support integrated prevention, although the magnitude of benefit from simultaneously changing several behaviors remains less certain than the associations observed in cohorts. Table 1 summarizes the evidence and distinguishes randomized intervention effects, intermediate risk-factor outcomes, and observational associations with clinical events.

## 15. Research Gaps and Future Directions

Despite compelling evidence supporting lifestyle modification, important evidence gaps remain. Long-term adherence is difficult, and many estimates of clinical events come from observational studies vulnerable to residual confounding, reverse causation, and exposure misclassification. Pragmatic randomized trials are needed, where feasible, to test sustainable multicomponent interventions and patient-important outcomes. Future studies should better distinguish physical activity, structured exercise, sedentary behavior, and cardiorespiratory fitness; examine resistance training and body-composition outcomes; and characterize sleep by duration, quality, regularity, timing, and sleep disorders. Alcohol research requires caution because observational associations should not be translated into recommendations to initiate drinking. Precision lifestyle medicine, including wearable technologies and improved phenotyping of adiposity and muscle health, may help tailor prevention strategies, but implementation should remain equitable and evidence-based [62].

## 16. Conclusions

Modifiable health behaviors remain fundamental to cardiovascular prevention. The strongest practical recommendations are to avoid tobacco exposure, follow a high-quality dietary pattern, engage in regular physical activity that includes aerobic and muscle-strengthening exercise, reduce prolonged sedentary time, and promote healthy multidimensional sleep. Evidence strength differs across domains: randomized trials support several dietary and exercise effects on cardiovascular events or risk factors, whereas many long-term estimates for sleep, sedentary behavior, alcohol, and composite lifestyle scores are observational. Although observational evidence suggests that low to moderate alcohol could have some cardiovascular benefit, it has not been demonstrated in a randomized controlled trial to prevent or reduce cardiovascular outcomes.

Integrated prevention should also distinguish health behaviors from clinical health factors. Life’s Essential 8 appropriately combines both domains, but body weight, blood pressure, glucose, and lipids are not lifestyle behaviors themselves. Weight management should account for central adiposity, skeletal muscle mass, strength, and function, particularly with aging. Future research should prioritize rigorous causal inference, pragmatic intervention trials, long-term adherence, and implementation strategies that translate evidence-based lifestyle interventions into routine clinical care and public health practice.

## Figures and Tables

**Table 1:** Major modifiable lifestyle factors in cardiovascular disease prevention: mechanisms, selected evidence, quantitative findings, and clinical implications.

Lifestyle Factor	Principal Mechanisms or Pathways	Key Evidence (Selected Studies)	Quantitative Findings	Clinical Implications
Healthy Dietary Patterns	Improved lipid profile, blood pressure, glycemic control, and endothelial function; reduced inflammation and oxidative stress.	PREDIMED randomized trial [10]; DASH and DASH-Sodium randomized trials [13,14]; Million Veteran Program prospective cohort [20]; AHA dietary guidance [8].	PREDIMED: MACE HR 0.69 (95% CI 0.53–0.91) with extra-virgin olive oil and HR 0.72 (0.54–0.95) with nuts, each versus advice to reduce dietary fat [10]. DASH: SBP/DBP lower by 5.5/3.0 mmHg versus control [13]. MVP: each 10-point higher hPDI associated with lower total CVD risk (HR 0.84; 95% CI 0.80–0.88) [20].	Emphasize whole, minimally processed foods and overall dietary quality. Prioritize vegetables, fruits, legumes, whole grains, nuts, and unsaturated fats; limit sodium, refined carbohydrates, and processed meats.
Smoking Cessation and Avoidance of Secondhand Smoke	Reduced thrombosis, inflammation, and oxidative stress; improved endothelial function and reduced ongoing vascular injury.	CHANCES individual-participant-data meta-analysis [31]; HF meta-analysis of 26 prospective studies [34]; secondhand-smoke meta-analysis [35].	CHANCES: quitting 5–9 years previously versus continued smoking was associated with lower CV mortality (HR 0.84; 95% CI 0.73–0.95) [31]. HF: at 30 years after quitting, RR 0.72 (95% CI 0.57–0.90) versus current smoking [34]. Secondhand smoke: higher ischemic heart disease risk versus no exposure (RR 1.27; 95% CI 1.10–1.48) [35].	Recommend complete cessation at every age, provide behavioral and pharmacological support, and avoid secondhand smoke. The magnitude and time course of benefit vary with prior exposure and population.
Regular Physical Activity	Improved endothelial function, blood pressure, insulin sensitivity, lipid profile, and cardiorespiratory fitness; resistance exercise preserves muscle mass and strength.	Dose–response meta-analysis of 94 prospective cohorts [36]; AHA resistance-training scientific statement [37]; PURE prospective cohort [39].	Activity equivalent to approximately 150 min/week of moderate-intensity exercise, compared with inactivity, was associated with lower all-cause mortality (RR 0.69; 95% CI 0.65–0.73) and cardiovascular mortality (RR 0.71; 95% CI 0.66–0.77) [36]. The largest relative gains occurred when moving from inactivity to some activity.	Aim for at least 150 min/week of moderate-intensity or 75 min/week of vigorous-intensity aerobic activity, plus muscle-strengthening exercise at least twice weekly. Progress according to ability and clinical status [4,5,37].
Alcohol Consumption	Heterogeneous effects on lipids, hemostasis, blood pressure, and cardiac electrophysiology; higher exposure can cause myocardial toxicity. Favorable biomarker changes do not establish cardiovascular protection.	Meta-analysis of eight prospective studies [45]; National Academies evidence reviews [46]; pooled prospective and genetic analyses [48,49].	Light-to-moderate intake, defined as <14 drinks/week in the meta-analysis, was associated with lower HF risk versus nondrinking (RR 0.85; 95% CI 0.78–0.93) [45]. This observational association does not establish a causal benefit or a safe intake threshold.	Do not initiate alcohol for cardiovascular prevention. For those who drink, emphasize minimizing intake, avoiding binge drinking, and considering individual health risks.
Healthy Sleep	Regulation of sympathetic activity, inflammation, glucose metabolism, endothelial function and blood pressure; sleep disorders may adversely affect these pathways.	Prospective-study meta-analysis [51]; AHA sleep statement [52]; Life’s Essential 8 framework [50]; older-adult sleep and prefrailty cohort [54].	Short sleep: CHD RR 1.48 (95% CI 1.22–1.80); stroke RR 1.15 (1.00–1.31); total CVD RR 1.03 (0.93–1.15; not significant). Long sleep: CHD RR 1.38 (1.15–1.66); stroke RR 1.65 (1.45–1.87); total CVD RR 1.41 (1.19–1.68) [51]. Sleep-duration categories varied by study; estimates reflect associations.	Aim for 7–9 h/night for most adults, with consistent timing and good sleep quality. Evaluate sleep apnea and other sleep disorders; consider underlying illness or frailty when sleep is prolonged [50,52,53,54].
Reduced Sedentary Behavior	Increased skeletal-muscle activity and energy expenditure; potentially improved glucose regulation, lipid metabolism, and vascular function.	Meta-analyses of sedentary time and health outcomes [55,56].	High versus low sedentary time was associated with higher cardiovascular mortality (HR 1.18; 95% CI 1.11–1.26) [55]. Trials of sitting reduction primarily assess intermediate cardiometabolic outcomes; a specific reduction in cardiovascular events has not been established.	Minimize prolonged sitting, incorporate regular movement breaks, and replace sedentary time with activity where feasible, including among people who meet exercise targets.
Overall Cardiovascular Health: Life’s Essential 8	Integrates four health behaviors with four clinical health factors, capturing multiple vascular and metabolic pathways.	AHA Life’s Essential 8 framework [50]; systematic review and meta-analysis of 34 observational studies [57].	Higher versus lower LE8 scores were associated with lower CVD risk (HR 0.47; 95% CI 0.39–0.56), all-cause mortality (HR 0.54; 95% CI 0.43–0.69), and cardiovascular mortality (HR 0.37; 95% CI 0.26–0.52), with substantial between-study heterogeneity [57].	Address diet, activity, nicotine exposure, and sleep alongside body weight, blood pressure, glucose, and lipids. Consider central adiposity, muscle preservation, strength, and physical function when managing weight [50,58].

AHA = American Heart Association; BMI = body mass index; CVD = cardiovascular disease; CV = cardiovascular; DBP = diastolic blood pressure; CHD = coronary heart disease; HF = heart failure; hPDI = healthful Plant-Based Diet Index; MACE = major adverse cardiovascular event; MVP = Million Veteran Program; RCT = randomized controlled trial; SBP = systolic blood pressure; LE8 = life essential 8; RR = relative risk; HR = hazard ratio; CI confidence interval.

## Data Availability

No new data were created or analyzed in this study. Data sharing is not applicable to this article.
